# Pathophysiology in Brain Arteriovenous Malformations: Focus on Endothelial Dysfunctions and Endothelial-to-Mesenchymal Transition

**DOI:** 10.3390/biomedicines12081795

**Published:** 2024-08-07

**Authors:** Jae Yeong Jeong, Adrian E. Bafor, Bridger H. Freeman, Peng R. Chen, Eun S. Park, Eunhee Kim

**Affiliations:** Vivian L. Smith Department of Neurosurgery, McGovern Medical School, The University of Texas Health Science Center at Houston, Houston, TX 77030, USA; jaeyeong.jeong@uth.tmc.edu (J.Y.J.); adrian.e.bafor@uth.tmc.edu (A.E.B.); bridger.h.freeman@uth.tmc.edu (B.H.F.); peng.r.chen@uth.tmc.edu (P.R.C.); eunsu.park@uth.tmc.edu (E.S.P.)

**Keywords:** arteriovenous malformations (AVMs), endothelial cells (ECs), endothelial dysfunction, endothelial-to-mesenchymal transition (EndMT)

## Abstract

Brain arteriovenous malformations (bAVMs) substantially increase the risk for intracerebral hemorrhage (ICH), which is associated with significant morbidity and mortality. However, the treatment options for bAVMs are severely limited, primarily relying on invasive methods that carry their own risks for intraoperative hemorrhage or even death. Currently, there are no pharmaceutical agents shown to treat this condition, primarily due to a poor understanding of bAVM pathophysiology. For the last decade, bAVM research has made significant advances, including the identification of novel genetic mutations and relevant signaling in bAVM development. However, bAVM pathophysiology is still largely unclear. Further investigation is required to understand the detailed cellular and molecular mechanisms involved, which will enable the development of safer and more effective treatment options. Endothelial cells (ECs), the cells that line the vascular lumen, are integral to the pathogenesis of bAVMs. Understanding the fundamental role of ECs in pathological conditions is crucial to unraveling bAVM pathophysiology. This review focuses on the current knowledge of bAVM-relevant signaling pathways and dysfunctions in ECs, particularly the endothelial-to-mesenchymal transition (EndMT).

## 1. Introduction

Brain arteriovenous malformations (bAVMs) are a tangle of blood vessels caused by an abnormal direct connection between arteries and veins bypassing the normal intervening capillary bed. Although bAVMs are rare (~18/100,000 population) [1,2], the ambiguous nature of abnormal vessels (the AVMs are not typical arteries nor veins) means that they are fragile and prone to rupture, which is a high risk of hemorrhagic stroke. Approximately 34–79% of bAVMs rupture, resulting in significant morbidity and mortality. In young patients (<50 years old), bAVMs are the leading cause of intracerebral hemorrhage (ICH) [3,4,5,6]. The treatments of bAVMs rely on surgical resection, embolization, or radiotherapy; however, these procedures carry their own risks of complications and can be limited by the bAVM size and location [7]. Even after a successful procedure, the bAVM can reoccur [8]. Currently, there are no pharmaceutical agents approved for the treatment of bAVMs, in part due to a poor understanding of bAVM pathophysiology. Elucidation of pathophysiologic mechanisms underlying bAVMs is, therefore, critical to developing pharmaceuticals to treat bAVM patients. Over the last two decades, studies using human samples and animal models have established the fundamental understanding that endothelial dysfunction, as a consequence of genetic mutations, is essential for bAVM development. In this review, we document recent advances in the understanding of bAVM pathogenesis and evolution, with a focus on underlying mechanisms of endothelial dysfunctions.

## 2. Genetic Mutations as the Cause of bAVMs

### 2.1. HHT Gene Mutations in bAVMs

Brain AVMs are usually congenital, which means that the patients are born with bAVMs. However, the majority of bAVMs are not inherited but are rather sporadic. Only around 5% of bAVM cases are associated with known hereditary conditions, such as hereditary hemorrhagic telangiectasia (HHT), which is also known as Rendu–Osler–Weber syndrome [9,10]. HHT affects approximately 0.01% of the populations (1 in 5000–8000) and is characterized by telangiectasias and arteriovenous malformations (AVMs) of the skin, brain, lung, and liver [9,10,11,12]. Around 10% of HHT patients have bAVMs, with a significantly higher prevalence seen in HHT type 1, HHT1 (13.4%) compared to those with HHT type 2, HHT2 (2.4%). bAVM prevalence in HHT is not found to differ based on sex, though it has been found that the sizes of bAVMs in HHT patients were typically small (86.9% of bAVM ≤ grade 2, by Spetzler-Martin grading method), and more than half (55.2%, 95% confidence interval (CI) [38.3%, 72.1%]) of these patients were symptomatic [13]. Pathogenesis of HHT1 and HHT2 involves germline mutations in transforming growth factor-β (TGF-β) signaling gene, such as Endoglin (*ENG*, also known as cluster of differentiation 105 (CD105), a type I transmembrane glycoprotein) and activin receptor-like kinase 1 (*ALK1*, also known as *ACVRL1*) [14,15]. The juvenile polyposis (JP)-HHT subtype is associated with mutations on gene encoding suppressor of mothers against decapentaplegic 4 (SMAD4) and accounts for approximately 2% of HHT cases [16,17,18,19,20]. The patients with JP-HHT often develop polyps in the gastrointestinal tract [21] and/or aortic dilation [22]. In comparison to *ENG* and *ALK1*, de novo mutations are identified at a higher rate in JP-HHT families [19,23]. In addition, mutations on growth differentiation factor 2 (*GDF2*) (also known as bone morphogenetic protein 9 (*BMP9*)) have been rarely (<1%) detected in HHT-like syndromes, which are distinguished by patients suffering from recurrent epistaxis, telangiectasia, and other symptoms associated with HHT, along with a family history of similar complaints [20,24,25,26]. This suggests that the *GDF2* mutation could be involved in the pathogenesis of HHT. With the germline mutations, several studies reported de novo mutations or somatic mosaicism of the HHT genes, *ENG* and *ALK1* [27,28,29,30,31], as well as *SMAD4* [19,23] in local tissues, including peripheral blood and hair bulbs. The somatic mutations existed in conjunction with the germline mutations, suggesting that bi-allelic loss of *ENG* or *ALK1* is necessary for the development of vascular malformations in HHT [32]. In mouse models, *ENG* or *ALK1* loss of function mutation with overexpression of vascular endothelial growth factor (VEGF) promoted bAVM formation [33,34]. Although the HHT-related bAVM comprise a relatively small fraction of the total bAVMs, the findings using animal models of bAVMs generated by HHT-gene mutations provide valuable insights into bAVM pathophysiology.

### 2.2. Somatic Mutations in bAVMs

More than 95% of bAVM cases are sporadic, without a family history of the disease, suggesting the involvement of non-inheritable factor(s). Accordingly, advanced genomic sequencing techniques identified several somatic mutations in the local bAVM tissues. A recent study found a high frequency of Kirsten rat sarcoma virus (KRAS) mutations in sporadic bAVMs. Nikorav et al. discovered that more than 60% of sporadic bAVMs (45 of the 72 patients) had one of the activating KRAS mutations, including p.Gly12Asp (G12D), p.Gly12Val (G12V), p.Gly12Cys (G12C), and p.Gln61His (Q61H). The study confirmed that the associated mutations were undetectable in 21 paired blood samples [35]. Following studies consistently detected the KRAS mutations in up to 72% of bAVM tissues, additionally identifying further KRAS mutations such as Gly12Ala (G12A). Overall, KRAS G12D and G12V were the most prevalent mutations detected in these bAVMs [36,37,38,39,40]. In addition to the KRAS mutations, mosaic variants in genes, such as Neuroblastoma RAS (*NRAS*), v-raf murine sarcoma viral oncogene homolog B1 (*BRAF*)*,* and mitogen-activated protein kinase kinase 1 (*MAP2K1*, also known as *MEK1*) were also discovered in sporadic intracranial and extracranial vascular malformations. These variant allele frequencies were prevalent in up to 26% of the affected tissue, with an increased frequency in high flow AVMs. No variant alleles were found in paired blood samples [36,41]. Additionally, preclinical studies using animal models validated the pathogenicity of the mutations on KRAS, BRAF, and MEK1 by demonstrating the development of abnormal vessels when these local mutations were induced in mice or zebrafish [41,42,43]. A further preclinical study also exhibited that Harvey RAS (HRAS) mutation induced vascular malformations accompanied with hemorrhages [44].

### 2.3. Phakomatoses and bAVMs

Phakomatoses are a heterogenous group of congenital neurocutaneous disorders that include Neurofibromatosis (types 1 and 2), Tuberous Sclerosis, Von Hippel–Lindau, and many others [45]. Of the phakomatoses, a select few are associated with bAVMs, including Sturge–Weber syndrome (SWS) and associated conditions, as well as Wyburn-Mason Syndrome (WMS). SWS, also called encephalofacial angiomatosis, is a neurocutaneous disorder characterized by congenital capillary malformations (CMs) and angiomas of the face, choroid, and/or leptomeninges, resulting in treatment-refractory seizures, glaucoma, and the classic facial nevus flammeus (also called a “port-wine stain”), typically occurring in a trigeminal nerve distribution [46]. This condition is quite rare, affecting approximately 1 in 20,000 to 1 in 50,000 live births [47]. Activating somatic mosaic mutations in the guanine nucleotide-binding protein subunit alpha q (GNAQ) and GNA11 genes have been implicated in the pathogenesis of SWS. A study using whole-genome sequencing from affected tissue samples first identified the role of non-synonymous single-nucleotide mutations in GNAQ (found at the 9q21.2 locus of chromosome 9) in SWS, as well as non-syndromic port-wine stains [48]. Another study later confirmed these findings, demonstrating enriched GNAQ mutations in ECs of CM, with GNAQ p.R183Q being the most common [49]. GNAQ encodes for Gαq, which is the alpha subunit of the heterotrimeric guanosine triphosphate (GTP)-binding proteins (G proteins) that function by linking with G-protein coupled receptors (GPCRs) to activate various intracellular signaling cascades [50]. The p.R183Q mutation has been shown to increase extracellular signal-regulated kinase (ERK) activity, while p.Q209L mutations result in hyperactivation of the mitogen-activated protein kinase (MAPK) and Hippo/Yes-associated protein (YAP) pathway, promoting disorganized vascular development and oncogenesis [48,51]. A more recent study also found that mosaic GNA11 mutations also play a causative role in the phenotypic spectrum of SWS [52]. GNA11 is a homologue of GNAQ, encoding for a different Gα subunit (Gα11) [47]. Kippel–Trenaunay syndrome (KTS) and Parkes Weber syndrome (PWS), sometimes considered together under the umbrella Kippel–Trenaunay–Weber syndrome (KTWS), are rare phakomatosis variants often grouped in the SWS family. KTS is characterized by a port-wine stain limited to part of an extremity, limb hypertrophy, or venous varicosities, whereas PWS mainly differs from KTS by the added presence of detectable AVMs [53]. While the pathogenesis of these syndromes remains unclear, KTS is associated with mutations in the phosphatidylinositol-4,5-bisphosphate 3-kinase catalytic subunit alpha (PIK3CA) gene, which encodes for the catalytic subunit of the phosphatidylinositol 3-kinase (PI3K) enzyme responsible for activating the protein kinase B (AKT)-mammalian target of rapamycin (mTOR) signaling cascade [54]. In contrast, PWS and the closely related CM-AVM disorder are caused by autosomal-dominant, inactivating heterozygous germline mutations in the RAS p21 protein activator 1 (RASA1) gene [55,56]. The RASA1 gene encodes the p120-RasGTPase-activating protein (p120-RasGAP), which negatively regulates the Ras/MAPK-signaling pathway [57]. WMS, also known as Bonnet–Dechaume–Blanc syndrome, is a nonhereditary phakomatosis characterized by AVMs of the face, retina, and brain [58]. bAVMs in WMS often localize to the midbrain region and are prone to rupture, posing a significant mortality risk for these patients. This condition is exceptionally rare, with fewer than 100 cases reported in the literature since first being formally identified in 1937 [58,59]. Consequently, very little is known about the etiology, and there are no known risk factors or genetic causes for this condition. It has been theorized that early insult of neural crest cells and cephalic mesoderm migration during organogenesis may be the cause [59]. Further investigation into the mechanisms causing abnormal vascular development in this condition may provide valuable insights into conserved mechanisms between WMS and non-WMS bAVMs, promoting a deeper understanding of the pathogenesis of this disease.

## 3. Endothelial Cells as the Major Player in bAVM Pathology

**Figure 1:**
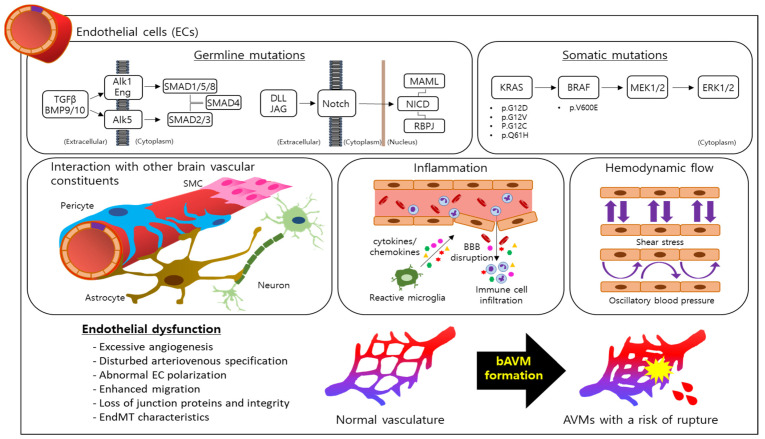
Schematic overview of the factors related to endothelial dysfunction in bAVM pathogenesis. Germline or EC-specific somatic mutations trigger EC dysfunction, which is influenced by various factors, including other vascular and immune cells, humoral factors such as inflammatory cytokines/chemokines, and hemodynamic flow. The direct or indirect interactions with these factors orchestrate EC dysfunction featured by disturbed polarization and migration, acquiring mesenchymal features, loss of junction proteins, and excessive angiogenesis, leading to abnormal vascular development and remodeling, as well as impairment of vessel integrity, subsequently resulting in brain arteriovenous malformations.

The endothelium, a single layer lining inner blood and lymphatic vessels, is comprised of endothelial cells (ECs). ECs play several key roles in the maintanence of tissue homeostasis by promoting the distribution of oxygen and nutrients, regulating the immune response via interactions with immune cells and chemokines/cytokines, as well as assisting in the regulation of blood flow [60]. ECs are highly flexible and adaptive, influenced by the surrounding microenvironment to acquire their specialized molecular characteristics based on physiological and pathological circumstances (e.g., hemostatic balance, age, inflammation, disease, etc.) [61,62,63]. While dysregulation of angiogenesis and various vascular functions contribute to a variety of cerebrovascular diseases, the pivotal roles of ECs are highlighted in bAVMs. Studies have shown that a robust development of bAVMs were induced in mice by deletion of ENG or AlK-1 specifically in ECs [33,34], but not in pericytes or macrophages [34,64]. It is also confirmed that mutations in bone marrow derived ECs or brain ECs are sufficient to induce bAVM development in mice [43,65]. This evidence demonstrates that the EC-specific gene mutations play a pivotal role in bAVMs. A series of complex cellular and molecular phenomena are triggered by the mutations and orchestrate EC dysfunctions and bAVM development (Figure 1). However, the exact mechanisms by which these gene mutations induce the EC dysfunction underlying bAVM pathophysiology has yet to be fully explained.

### 3.1. Molecular Signaling in ECs Relevant to bAVMs

#### 3.1.1. TGFβ/BMP Signaling

**Figure 2:**
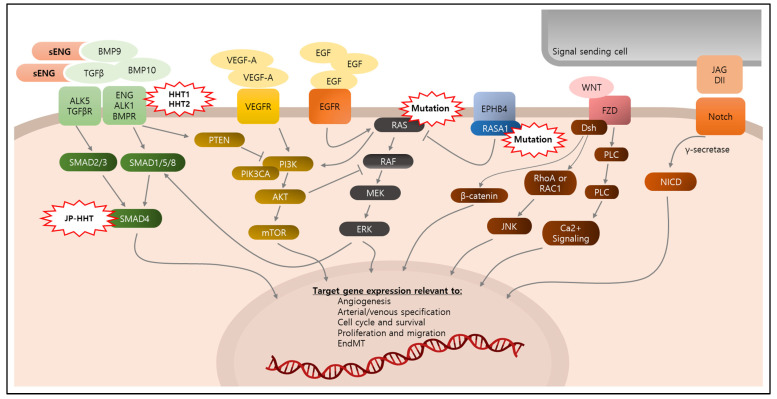
Simplified scheme of signaling pathways in ECs relevant to bAVM pathogenesis. Activation of SMADs in ECs can occur via ligand (BMP9/10, TGFβ) binding to the TGFβ receptor complexes. Loss-of-function mutations on ENG and ALK-1 gene, the co-receptors of TGFβR and BMPR, and SMAD2, are the cause of HHT syndromes. Circulating sENG can act as a decoy receptor for the ligands of BMPs in TGFβ signaling, which is implicated in bAVM development. VEGFR2 binding with VEGF-A activates PI3K/AKT/mTOR signaling, which can be inhibited by PTEN, an effector of TGFβ signaling. The RAS/RAF/MEK/ERK pathway, a straightforward signaling cascade to regulate various cellular functions. RAS can activate PI3K, whereas AKT inhibits RAF. Several activating somatic KRAS mutations were found in human sporadic bAVMs. EGFR binding with EGF can also activate RAS signaling. Activating somatic KRAS mutations detected in human sporadic bAVMs are sufficient to induce bAVMs. RASA1 interacting with EPHB4 is the endogenous inhibitor of RAS, and mutations on the RASA1 gene have been implicated in low-flow AVM formation. Canonical (Dsh-mediated) WNT signaling, non-canonical (Ca^2+^-dependent) WNT signaling, and Notch pathways are also involved in bAVM pathological mechanisms through regulation of EndMT and vascular specifications. Evidence suggest that these pathways cross-communicate with TGFβ signaling. The listed signaling pathways regulate their target gene expression involved in EC functions (cell cycle, survival, proliferation, and migration), EndMT, vessel specification, and angiogenesis. Abnormal activation/suppression of these pathways by germline or somatic mutations is relevant to vascular malformations.

Among various signaling pathways and molecules involved in bAVMs (Figure 2), transforming growth factor-β (TGFβ) and the BMP superfamily are the most critical in bAVM pathophysiology. Involvement of the TGFβ/BMP superfamily has been shown in EC biological processes, including in modulation of EC quiescence, vessel diameter, and arteriovenous specification [66,67,68,69,70,71]. EC-specific deletion of TGFβ signaling can induce increased vulnerability of cerebrovascular integrity, along with increased EC proliferation and defective pericyte recruitment [72]. The HHT-related molecules, ENG and ALK1, are co-receptors of TGFβ and BMPs, and function by constructing the receptor complex with the TGFβ receptor [14,15]. Briefly, ENG binds with TGFβ type I and II receptors (including ALK1 and ALK5) [73,74,75]. BMP9 has a high affinity to bind to ENG and ALK1 in the receptor complex, leading to phosphorylation of SMAD1/5/8, and then bind with activated SMAD2/3. The activated SMADs form a heteromeric complex with SMAD4, which is then translocated into the nucleus where it regulates transcription of target genes associated with vascular responses [76,77,78,79,80,81,82].

ENG is predominantly expressed in ECs and endothelial colony-forming cells (ECFCs) [73,74,75]. During development, ENG is expressed throughout the endothelium; however, in adults, ENG expression is mostly restricted to the quiescent arterial endothelium, which can be re-expressed under endothelial stress [79,83]. Global deletion of ENG results in arterial and venous endothelial abnormalities and defective vasculature remodeling, wherein blood vessels fail to remodel from a primitive plexus to a segmental and branched vasculature. Additionally, visible hemorrhages and impaired vascular smooth muscle development were shown in ENG-deleted animal embryos [84,85]. Moreover, in the neonatal retina, endothelial-specific Cre-inducible ENG deletion increased EC proliferation, delayed remodeling, and reduced migration of the newly formed capillaries, resulting in poor vascular remodeling and abnormal vessel formation [86]. In retinal ECs, ENG was shown to be haplosufficient in the mediation of retinal vasculature development and angiogenesis via both SMAD and MAPK signaling pathways [69]. Another study showed that though ENG heterozygous mice developed normally, older animals displayed vessel wall defects, such as excessive vessel branching and enhanced pericyte and vascular smooth muscle cell (SMC) coverage [87]. In addition to the membrane bound form, ENG may circulate in a separate form as soluble ENG, which is produced by matrix metalloproteinase 14 (MMP-14) cleavage of the ENG C-terminal [88,89]. Soluble ENG can function as a decoy receptor of circulating TGFβ and BMP9, leading to reduced activation of the TGFβ pathway [90,91]. Increased soluble ENG is involved in the pathogenesis of preeclampsia in patients with hypertension, diabetes or certain type of cancers [91,92,93,94]. It has also been shown that soluble ENG contributes to development of dysplastic vessel formation in mice [95,96]; however, the exact role of soluble ENG in bAVMs still requires further investigation.

ALK1 also plays a key role in angiogenesis. In the vascular development and remodeling process, ALK1 expression is limited to the arterial endothelium, but it can be induced during angiogenesis following inflammation or wound healing [66,97,98,99,100]. Genetic deletion of *ALK1* in animal models resulted in early embryonic lethality, with defects found in the formation and remodeling of a functional vascular network. These animals also showed various deficits in vascular function, including diminished SMC recruitment and arterial EphrinB2 expression, suggesting ALK1 is required for determining certain vascular endothelial properties [101,102]. *ALK1* heterozygous mice showed age-dependent vascular lesions and gastrointestinal bleeding, similar to findings in HHT patients [103]. In the endothelial-specific *ALK1*-deleted mice, the lethality was delayed. However, these animals still displayed AVMs with poorly developed SMC layers within a grossly tortuous arterial system as well as hemorrhages [99]. Furthermore, *ALK1*-mutated zebrafish showed a loss of receptor function, resulting in an increased number of ECs and enlarged intracranial vessels, suggesting that ALK1 regulates EC proliferation and vessel diameter [104]. Of note, one controversial study later concluded that ALK1 loss has no effect on arterial EC proliferation in zebrafish embryos. However, this study found that EC migration was enhanced in the direction of blood flow, suggesting that ALK1 may play a role in the EC response to the flow dynamics [70]. The effect of ALK1 overexpression on AVM development was also investigated in ALK1- vs. ENG-deficient mice. Interestingly, endothelial-specific overexpression of ALK1, but not ENG, suppressed the development of AVMs in the wounded skin and developing retinas of both ALK-1- and ENG-inducible knockout mice through regulation of Notch, SMAD, and AKT signaling in ECs [105].

BMPs are circulating growth factors in blood. Among BMPs, BMP9 and 10 are the two major ligands showing high affinity to bind to ENG and ALK1 [106,107,108,109,110,111]. Studies implicate BMP9 and 10 in vascular remodeling and endothelial quiescence. Evidence proposes the role of BMP9 and 10 in suppression of EC proliferation/migration and VEGF-induced angiogenesis [108,111,112,113,114,115,116]. However, there is a controversial study that showed BMP9 induces EC proliferation [117]. The role of BMP9 and 10 in vascular malformations has been also investigated. BMP9 inactivation or deficiency did not lead any obvious defect in the vessels of the lymphatic system or retina [118,119,120,121]; however, the BMP9 deficiency resulted in defective lymphatic vessel formation and drainage [119,121]. In contrast to BMP9, BMP10-deficient mice developed cardiac and vascular defects and were embryonically lethal [118,122]. One study also found that temporal knock-down of BMP10 induced abnormal vessel phenotypes similar to those seen in ALK-1 defective mice, including enlarged arteries and AVMs with a higher number of ECs [68]. It also demonstrated that neutralizing BMP10 in BMP9-deficient mice inhibited vascular expansion and increased retina vessel density [118,120]. This evidence suggests that BMP10, rather than BMP9, is a key ligand for TGFβ signaling and plays a critical role in vessel development and/or remodeling.

#### 3.1.2. Notch Signaling

Notch proteins are a family of type 1 transmembrane receptor proteins that contribute to cell differentiation, proliferation, survival, and apoptosis. The majority of Notch signaling activations are induced by binding with integral cell surface proteins called DSLs (delta, serrate, and longevity assurance gene 2 (Lag2)) in the respective binding domain, leading to direct cell-to-cell interactions. The ligand binding induces cleavage of the Notch extracellular domain by an a-disintegrin-and-metalloprotease (ADAM), and the intramembrane domain by γ-secretase, releasing the Notch intracellular domain (NICD). The NICD then migrates into the nucleus where it forms a transcriptional activation complex with DNA binding proteins (e.g., Core binding factor 1 (CBF1), recombination signal binding protein for immunoglobulin kappa J region (RBPJ), Suppressor of Hairless (Su(H)), and Lag1), subsequently regulating target gene transcription [123,124,125]. During vascular development and remodeling, Notch signaling regulates arteriovenous specification, EC differentiation, and blood vessel sprouting and branching [126,127,128,129]. Notch signaling, in particular, induces a change in cell-cycle-regulator expression and promotes gap 1 (G1) arrest during the arterial specification stage [130,131]. Thus, Notch signaling plays a crucial role in the modulation of arterial fate specification and vascular patterning in the endothelium [128]. Furthermore, Notch signaling affects high-flow-specific endothelial features implicated in the modulation of EC selection in relation to hemodynamic flow [132].

Animal studies have revealed that altered Notch function is linked to vascular malformations. Notch1 homozygous deletion animals exhibited embryonic death with direct anastomosis between arterial and venous circulation, and Notch1 and 4 double-deleted mice exhibited synergistic effects, resulting in more severe vascular abnormalities [133,134]. Notch signaling predominantly mediates the EC fate specification, as evidenced by the loss of arterial features when Notch is suppressed, but the loss of venous qualities when Notch is activated [135]. The role of Notch signaling in AVMs development has also been reported [136,137,138,139]. Notch1 gain of function in ECs caused AVMs with increasing arterial diameter, whereas Notch1 loss of function caused AVMs with partial interruption and slowed growth of the dorsal aortae [137]. It has also been shown that EC-specific activation of Notch4 increased arterial characteristics in veins and caused bAVM shunts [140]. Conversely, the repression of Notch4 reorganized ECs and restored venous characteristics, which then lead to bAVM regression [138].

Furthermore, it has been demonstrated that the cross-communication between TGF and Notch signaling modulates EC activity during vessel formation and arteriovenous specification [141,142]. The BMP9-ALK1-SMAD signaling cascade activated downstream of Notch signaling [143], and Alk1 overexpression could alleviate the AVM phenotype by regulating the expression of Notch and SMAD target genes [105]. Moreover, deficiency of Tmem100, a gene functionally regulated by Alk1-BMP signaling, resulted in embryonic lethality with disorganized arteries and downregulated Notch and AKT signaling [144]. Therefore, it is conceivable that there is a functional cross-communication system between Alk1-BMP signaling and Notch signaling in the development of arterial endothelium. These findings point to a pivotal link between Alk1 and Notch signaling during vascular development.

#### 3.1.3. RAF/MEK/ERK Signaling

RAS oncogene family consists with KRAS, HRAS, and NRAS. Although they have a significant sequence homology, each RAS family member is expressed in different pattern, depending on the tissue type, and has a distinctive role in cellular signaling pathways [145]. The causal role of RAS mutations in abnormal vascular development in animal models suggests that activation of RAS signaling pathways should be critical in bAVM development [41,42,43,44]. Among the RAS mutations, KRAS mutations were highly detected in human bAVMs [35,38]. The KRAS genes code for a protein that belongs to the small GTPase superfamily, which enables the catalyzation of the hydrolysis of GTP into guanosine diphosphate (GDP). Binding with GTP activates KRAS signaling to trigger the activation of downstream effector cascades, which mediate diverse cellular functions including cell proliferation, migration, and survival [146,147].

Among several downstream signaling effectors activated by KRAS, the RAF/MEK/ERK pathway seems most critical in bAVM development. The RAF/MEK/ERK signaling is a straightforward cascade, wherein KRAS-recruited RAF forms dimers with RAF family members, which activates MEK and ERK 1/2, sequentially. The activated ERKs translocate into nucleus and activate transcription factors regulating the expression of genes involved in various cell functions [148,149]. It is revealed that activation of the RAF/MEK/ERK pathway is critical in bAVM development. In addition to the observation of mutations on *BRAF* and *MEK1* in human bAVM tissues [41], bAVMs with KRAS mutations showed significantly increased phospho (p)-ERK levels, but not the other associated signaling molecules, such as AKT and p38 MAPK (p38) [35,42,43]. In addition, GNAQ/GNA11 appears to directly influence ERK signaling, whereas RASA1 directly regulates RAS activity, revealing the important connection between SWS and PWS, respectively, with this signaling pathway [48,52,55,150]. In mice and zebrafish, MEK inhibitors were shown to reduce mutant KRAS-induced bAVM pathology [42,43,151]; however, PI3K inhibition did not show efficacy on KRAS^G12V^-induced shunts in the zebrafish models [42]. There are a few case studies that demonstrate that treatment with a MEK inhibitor, trametinib, could mitigate extracranial AVMs [152,153]; however, further studies are required to identify if RAF/MEK/ERK inhibition is also beneficial in cerebral AVMs, as well as the exact mechanisms by which the RAF/MEK/ERK pathway is involved in bAVM pathophysiology.

#### 3.1.4. VEGF Signaling

Vascular endothelial growth factors (VEGFs) are a family of secreted polypeptides with a cysteine-knot structure. The VEGF family has emerged as the most important regulator of angiogenesis and is essential in embryonic vasculogenesis and angiogenesis in both health and disease [154]. Among the VEGF family, VEGF-A is the most abundant and important pro-angiogenic factor seen in humans and rodents. VEGF-A binds to VEGF receptors, specifically VEGFR2 (also called kinase insert domain receptor (KDR) or fetal liver kinase 1 (Flk1), primarily expressed in ECs), and controls angiogenesis and vascular homeostasis by regulating EC proliferation, remodeling, and regression [154,155,156]. Excessive angiogenesis is one of the important biological mechanisms implicated in bAVM development. Previous studies have shown the importance of VEGF in bAVM progression. Increased VEGF expression levels were reported in endothelial and subendothelial layers and in the adjacent cells of resected human bAVM specimens [157,158,159,160], as well as in circulation [161]. In preclinical mouse models, enhanced VEGF-A expression was observed in dissected KRAS^G12V^-induced bAVMs, which coincides with enhanced p-VEGFR2 in ECs of the abnormal vessels, suggesting this endogenous VEGF signaling is activated by the KRAS mutation [43]. Importantly, studies using mice mutated with HHT genes have shown that VEGFR2 expression is correlated with bAVM development [162], and VEGF-A was required to trigger the bAVM phenotypes [163,164,165]. In addition, VEGF inhibitors reduced bAVM development and associated pathologies [166,167]. These demonstrate that the activation of VEGF signaling plays a key role in bAVM development. In addition, studies have revealed the link between VEGF signaling and the other signaling pathways associated with bAVM development. For example, the activation of TGFβ or ERK signaling stimulates VEGF expression, likely through regulation of hypoxia-inducible factor-1α (HIF-1α), a strong VEGF inducer [168,169,170,171,172]. On the other hand, VEGF is a strong activator of ERK in ECs [173], suggesting possible bifurcate functions in both up- and down-stream signaling cascades between RAS and VEGF signaling. Through further assessment of the effect of VEGF inhibition in bAVM development and bAVM-associated hemorrhages, an understanding of the interplay between VEGF signaling and the other associated signaling pathways would be essential to unveiling the bAVM pathophysiology.

#### 3.1.5. PI3K/AKT/mTOR Signaling

The PI3K/AKT/mTOR signaling cascade is an important effector pathway in angiogenesis that is primarily controlled by a variety of growth factors, such as fibroblast growth factor (FGF), platelet-derived growth factor (PDGF), and VEGF [150]. PI3Ks are a family of heterodimer lipid kinases recruited to the cell membrane after ligand binding with the respective growth factor receptor protein tyrosine kinase (GFRTK) occurs [174]. Through a series of subsequent processes, an important independent regulator of cell survival and cell cycle progression, AKT, is recruited [174]. AKT has been shown to promote cell survival through the inactivation of pro-apoptotic factors (e.g., procaspase-9, Fas-ligand), increased resistance to tumor necrosis factor (TNF)-related apoptosis, and upregulation of survival factor nuclear factor κB (NFκB) [175,176,177,178]. AKT then upregulates mTOR complex 1 (mTORC1) activity directly, via phosphorylation of the Protein-Rich AKT Substrate of 40 kDa (PRAS40), and indirectly, through phosphorylation of the Tuberous Sclerosis Complex (TSC) 2 (aka tuberin) [179]. Interestingly, the Phosphatase and Tension Homologue (PTEN), which is under negative regulation by TGFβ/BMP, has been shown to block AKT activation, revealing an important connection between the TGFβ/BMP and PI3K/AKT pathways [71]. mTOR complex 2 (mTORC2) is a rapamycin-insensitive complex that is poorly understood, though its ability to directly activate AKT is well known [180]. Though the PI3K/AKT/mTOR1C pathway has been well studied as the canonical pathway of mTOR activation, several AKT-independent pathways for mTOR activation have also been recognized. Of note, RAS/MEK/ERK signaling has been found to directly upregulate mTORC1 activity through AKT-independent phosphorylation of TSC2 by ERK 1/2, revealing an important mechanism for crosstalk between the RAS/RAF and PI3K/AKT pathways [181]. Furthermore, activated KRAS can directly activate the PI3K/AKT pathway by binding with the p110 subunit of PI3K [182]. Regardless of which preceding pathway is utilized, once mTOR1C undergoes activation, it then proceeds to act as a powerful promoter of cell-cycle progression, cell growth, and angiogenesis through the increased synthesis of many proteins [174].

Until recently, the direct connection between mTOR activity and bAVMs remained obscure. However, a study has now demonstrated that mTOR, p-mTOR, and fatty acid binding protein 4 (FABP4) expression were present in 70%, 55%, and 80% of ECs from human bAVM tissue, respectively [183]. In this study, the expression of these markers was notably absent in superficial temporal artery and cerebral vessels, suggesting that their role was linked to bAVM pathogenesis. As previously discussed, mutations in PIK3CA have been shown to cause KTS [54]. A recent case report found markers for both mTOR and RAS/MAPK pathway activation in the AVM tissues of a patient with simultaneous bAVM and peripheral AVM [184]. However, some evidence suggests that PI3K/AKT signaling may be more strongly associated with low-flow and venous malformations, as reported in this study [41]. Likewise, it has been reported that over half of sampled sporadic venous malformations possessed somatic tunica intima endothelial kinase 2 (TIE2) mutations [185]. TIE2 is a receptor tyrosine kinase, activated by ligands angiopoietin 1 (ANG1) and ANG2, that has been shown to play important roles in angiogenesis, vascular stabilization, and metastasis in various oncological, neovascular, and inflammatory processes through the activation of the PI3K/AKT/mTOR pathway [186]. Further still, a recent study modeling human bAVMs on a chip found that MEK inhibition (MEKi), but not PI3K inhibition, improved endothelial barrier function in samples containing KRAS^G12V^ mutations [187]. This finding suggests that KRAS^G12V^-mediated bAVMs utilize MEK/ERK signaling, not PI3K/AKT signaling, to induce pathology. However, the lack of vessel diameter reduction in response to MEKi in this study also supports the emerging theme that other signaling pathways or cell types are involved in KRAS-mutated bAVM pathogenesis, in addition to dysfunctional MEK/ERK signaling within ECs. It is clear that crosstalk between pathways must be considered in further investigation to correctly identify the precise mechanisms for bAVM pathogenesis and appropriate targets for treatment.

#### 3.1.6. Targeted Therapies in the Treatment of bAVMs

As knowledge expands regarding the many signaling pathways involved in bAVM pathogenesis, several novel pharmacologic agents have begun to be investigated for their potential to treat this disease (Table 1). Rapamycin, commercially known as sirolimus, is an antifungal metabolite that acts as an allosteric inhibitor of the mTOR complex 1 (mTORC1) after forming a gain-of-function complex with the 12-kDa FK506-binding protein (FKBP12) [188]. With recent discoveries regarding the involvement of mTOR signaling in certain bAVMs, such as in SWS, there has been a heightened interest into the efficacy of rapamycin in treating these conditions. For example, preliminary results from a European multicentric phase III clinical trial on the use of rapamycin in slow-flow vascular malformations revealed clinical improvement in 85% of patients, with the majority experiencing results within the first month of treatment [189]. Similarly, a pilot study using sirolimus in patients with SWS reported significant clinical improvements after 6 months of treatment [190]. Though use of this agent has been recently reported in the treatment of a number of inoperable bAVMs, yielding promising results, further investigation is needed to formalize the role of rapamycin as a safe and effective treatment for bAVMs [191]. Thalidomide is another potential therapy under investigation due to its immunomodulatory and anti-angiogenic effects [192]. This agent has been shown to stimulate platelet-derived-growth-factor-B (PDGFB) expression in ECs, promoting vessel maturation in HHT patients [87]. It was also shown to reduce vessel dysplasia and hemorrhages while increasing mural cell coverage and PDGFB expression in mouse bAVMs and ALK1-knockdown human brain ECs [193]. Another study using mice found that a metabolite of thalidomide possesses potent antiangiogenic activity in VEGF-induced corneal neovascularization [194]. A phase II clinical trial concluded that thalidomide is safe and very effective in treating recurrent epistaxis in HHT patients [195]. While these results are promising, further investigation is needed to determine the efficacy and safety of thalidomide as a treatment for human bAVMs. The anti-VEGF monoclonal antibody, bevacizumab, has been another drug of particular interest in the treatment of bAVMs. In fact, a single-arm clinical trial was recently published on the use of bevacizumab to treat human bAVMs, though it suffered considerable financial limitations [196]. Though no changes were reported in the bAVMs of two patients after 52 weeks, no major adverse effects were noted either [196]. However, this drug has previously been shown to reduce vessel density and dysplasia in the bAVMs of mice with a focal ALK1 deletion and human VEGF stimulation [167]. Bevacizumab has been recently shown to be efficacious in treating humans with severe extracranial AVMs, as well as in bleeding management for patients with HHT [197,198,199]. Due to these promising results, larger clinical trials should be organized to investigate the use of bevacizumab in treating bAVMs. Pazopanib is a multiple kinase inhibitor with potent antiangiogenic effects via inhibition of VEGF, PDGF, and FGF receptors, as well as CD117 (also known as stem cell factor receptor) [200]. This drug is currently under investigation in a US phase II/III clinical trial (NCT03850964) for its use in treating epistaxis and anemia in HHT patients. Another phase II clinical trial suggested potential efficacy of pazopanib in treating CNS hemangioblastomas in patients with Von Hippel–Lindau disease [201]. However, little is known about the use pazopanib in treating bAVMs, though further investigation is warranted. As previously mentioned, trametinib is an allosteric inhibitor of the MEK 1 and 2, leading to downstream disruption of MAPK signaling [202]. This drug has shown great promise in recent years towards the treatment of both extracranial AVMs and bAVMs, especially those harboring mutations in the KRAS and RAF/MEK/ERK axis. For example, a recent study demonstrated that trametinib effectively inhibited bAVM growth in mouse bAVMs harboring viral-mediated KRAS^G12V^ mutations [43]. There is also a US phase II clinical trial (NCT04258046) currently investigating the use of trametinib in treating complicated extracranial AVMs. Likewise, a European phase II clinical trial is also evaluating the use of trametinib in treatment-refractory AVMs or in those where the standard treatment is contraindicated (https://www.clinicaltrialsregister.eu/ctr-search/search?query=2019-003573-26 (accessed on 1 August 2024)). A pilot study (NCT06098872) is also currently investigating the use of trametinib in surgical unruptured bAVMs. With numerous studies showing the effectiveness of trametinib in treating AVMs, and further currently underway, continued efforts towards evaluating the role of trametinib in treating bAVMs will be of great benefit.

### 3.2. EC Dysfunctions in bAVMs

#### 3.2.1. Vascular Development and Arteriovenous Specification

Vascular development occurs through vasculogenesis and angiogenesis and is tightly controlled by several signaling pathways and transcriptional factors [210,211,212]. The de novo primitive vascular network is formed when ECs arise from mesodermal endothelial progenitor cells (EPCs, often referred to as angioblasts). The differentiation of ECs from EPCs is regulated by fibroblast growth factor 2 (FGF2) and BMP4 [213,214]. VEGF and Notch signaling are crucial in arteriovenous specification. Particularly, increased VEGF and Notch signaling lead to arterial differentiation [215,216,217,218]. Blood-flow-enhanced shear stress induces Notch signaling activation and gap junction α4 protein (connexin 37) expression in ECs, which subsequently induces EC cycle arrest and the expression of arterial specification genes [130]. Additional studies have shown that activation of VEGF-ERK signaling is also involved in this cell-cycle arrest and arterial specification [219,220,221]. In contrast, venous specification is regulated by reduced activation of VEGF signaling and chicken ovalbumin upstream promoter (COUP) transcription factor 2 (COUP-TFII, also known as nuclear receptor subfamily 2 group F member 2 (NR2F2)). COUP-TFII enhances cell cycle gene expression and vein-specific gene expression. Increased phosphoinositide 3-kinase (PI3K) signaling and expression of COUP-TFII were shown to be required for venous development [211,222]. A study using mouse and fish embryos showed that BMP-SMAD signaling directly induced venous gene expression [223]. It was also shown that deletion of *Smad4* or *Bmpr1a* gene in mouse ECs suppressed vein development [224].

After vasculogenesis, existing blood vessels further expand and remodel through angiogenesis, the formation of new vessels from preexisting ones. VEGF and Notch signaling also regulate angiogenesis; overall, angiogenesis is activated by VEGF but restricted by Notch signaling [225,226,227,228,229,230,231,232,233,234,235]. Specification of ECs as tip cells (ECs leading a vessel sprout) is induced by low Notch activity, whereas proliferation of stalk cells (following tip cells) is associated with higher Notch1 activity [230,236,237]. VEGF-A- and VEGFR2-deficient mice are embryonically lethal, due to a lack of EC differentiation, blood-island formation, and vasculogenesis [238,239,240]. Deficiency of another receptor of VEGF-A, VEGFR1 (also known as Flt1) also results in embryonic death as a consequence of EC overgrowth and vascular disorganization, suggesting that VEGFR1 is also essential for early vascular development [241]. A recent single-cell RNA sequencing (scRNA-seq) analysis has identified six key clusters in ECs (distinguished by *CLDN5* (gene encoding claudin-5) and *PECAM1* (gene encoding platelet endothelial cell adhesion molecule) in the ECs of normal human vessels, which are comprised of three clusters in arterial zonation as well as clusters of endothelium in arteries (*VEGFC*+, gene encoding VEGF-C), capillaries (*MFSD2A*+, gene encoding major facilitator superfamily domain-containing protein 2), and veins (*ACKR1*+, gene encoding atypical chemokine receptor type-1)) [242]. However, the detailed mechanisms by which EC heterogeneities impact EC differentiation/function and vessel formation still require further clarification.

#### 3.2.2. EC Polarization and Migration

EC expansion initially occurs in veins, and the ECs are recruited to arteries during vascular development [232,243,244]. Through lineage-labelling techniques, recent studies observed that ECs migrate from veins towards arteries in developmental retina vasculature, but the migration pattern was not seen in matured vessels [243,245,246]. This EC migration pattern is the result of innate programmed systems in ECs, directing them to move against the direction of flow [232,247,248]. TGFβ signaling seems critical to regulating the EC migration based on the studies showing that defective ALK1, ENG, or SMAD4 inhibits the blood-flow-guided EC migration [70,249,250]. In contrast, overexpression of ENG led ECs to become over-responsive to blood flow and resulted in excessive accumulation of ECs in arteries [249]. Similar overactivation of ECs against flow was shown by overexpression of Dach1 (endoding dachshund homologue 1), which was accompanied by increased artery branching [251,252]. It is also reported that loss of EC Wingless-related integration site (Wnt) family member 5A (Wnt5A) and Wnt11 resulted in similar overactivation of ECs in response to flow [253].

Cell polarization, the asymmetric distribution of cellular components, is important in cell migration and sprouting. Studies have shown that impaired angiogenesis was induced by disturbing EC polarization with modulating molecules, such as endophilin-A2, macrophage stimulating 1 (MST1), forkhead box protein O1 (FOXO1) cascade, the non-catalytic region of tyrosine kinase adaptor protein 1 (Nck1) and 2, or cell division control protein 42 (Cdc42) [254,255,256,257]. For the against-flow EC polarization and migration in zebrafish models, Wasb (the homologue of human Wiskott–Aldrich syndrome protein, a cytoskeletal regulator) or activated Notch signaling was required [258,259,260]. Decreased against-flow EC polarization and migration promoted regression of a subset of microvessels within the growing vasculature [261].

#### 3.2.3. EC Junction Integrity and Vessel Stability

Intact endothelial junctions provide blood vessel integrity and stability through regulating vascular permeability and leukocyte trafficking. The junction integrity is controlled by adhesion molecules including adherens, tight, and gap junctions [262,263]. In the blood–brain barrier (BBB), the junctional complexes between ECs form an interdependent network of brain ECs, as well as the other perivascular cells. Thus, the alteration of the junction structures and functions significantly affects EC properties, BBB integrity and permeability and may be involved in vascular remodeling [264].

Adherens junctions contain cadherins, Ca^2+^-dependent transmembrane proteins, and mainly control the passage of large molecular weight components. Vascular endothelial (VE)-cadherin is highly specific to ECs and is considered the most important adherens molecule for EC junctions [262,265]. In brain ECs, VE-Cadherin is the predominant adherens junction protein, though low levels of neural (N)- and epithelial (E)-cadherins have also been found [266,267]. VE-cadherin helps ECs to sense hemodynamic changes and activates a variety of signaling pathways through the formation of a complex with platelet endothelial cell adhesion molecule 1 (PECAM-1), along with VEGFR2 and VEGFR3 [263,268]. In mice, the absence of the VE-cadherin gene resulted in impaired VEGF-induced EC survival and angiogenesis, subsequently leading to defective vascular system development in mice [269]. Additional studies have shown that inhibition of VE-cadherin was sufficient to disturb cultured EC layers in vitro [270] and impaired vascular integrity and permeability in vivo [271,272,273,274]. This evidence supports the understanding that VE-cadherin is a critically important adhesion molecule in the EC junction. Moreover, impaired adherens junctions were observed in vascular malformations. In vivo and in vitro models of cavernous malformations, created by inducing cerebral cavernous malformation (CCM) gene ablation, showed disorganized VE-cadherin and β-catenin, leading to impaired cell junctions [275,276,277,278]. KRAS^G12D^ down-regulated VE-cadherin in in vitro cultured ECs [35,279], suggesting that the loss of VE-cadherin might be related to the bAVMs harboring KRAS mutation, making them prone to rupture [38]. Nectins are also a family of Ca^2+^-independent cellular adhesion molecules [280,281]. While the majority of investigation into their roles has been focused on nervous system and cancer immunology, some in vitro studies demonstrated that, among nectin members, nectin-2 and 3 were involved in EC junction integrity [282,283,284,285]. However, the role of nectins in brain ECs still remains largely unknown. Other studies have identified novel interactions involving EC adherens junctions, such as Ras-related C3 botulinum toxin substrate 1 (Rac1)-Guanine Nucleotide Exchange Factor (GEF) Trio, G-Protein α13, epidermal growth factor receptor kinase Substrate 8 (EPS8), YAP, heart-of-glass 1 (HEG1), and Ras-interacting Protein 1 (Rasip1), which were well-documented in a review by Lampugnani et al. [286].

Tight junctions regulate the passage of ions and molecules smaller than 800 dalton and are more prevalently found in the BBB [287,288]. Tight junction proteins include claudins, occludins, and an Immunoglobulin G (IgG)-type protein called junctional adhesion molecule (JAM) [289]. Claudins are the predominant tight junction protein found in all organ vessels, with more than 24 isoforms [289,290]. Among them, claudin-5 is the most important isoform in the brain ECs [291,292,293]. Claudin-5-deficient mice showed brain edema and disrupted BBB integrity, which could not be rescued by the other claudins (claudin-1, 3, and 12), resulting in death shortly after birth [292]. Claudin-3 is also constitutively expressed at the BBB. Although the expression levels are low in adult brain ECs [291,293], claudin-3 might play a role in BBB development and maintenance through interaction with Wnt/β-catenin signaling [294,295]. Occludin is the first tight junction protein to be described; however, its deficiency did not cause any obvious vascular impairment [296]. The literature suggests that occludins play regulatory roles in brain ECs by interaction with claudins and/or scaffolding proteins, such as zonular occludens (ZO)-1 and 2 and filamentous (F)-actin, rather than a role in cellular adhesion [297,298,299,300,301,302]. Another class of tight junction molecules are JAMs, a family of 2 Immunoglobulin-domain proteins. In ECs, JAM-A, JAM-B, and JAM-C, and the related EC-selective adhesion molecule (ESAM) have been identified, although these proteins are not specific for ECs [303,304,305,306]. The JAMs are considered key molecules in establishing tight junction complexes and tubule formation to support EC barriers and leukocyte extravasation [304,307,308,309,310,311]. In addition, ESAM is a newly identified transmembrane junction protein that has similar structure to JAMs [312,313,314]. While its role remains to be identified, studies showed that ESAM is a marker for hematopoietic progenitors and is involved in EC interaction, potentially as a regulator of vascular development and neutrophil trafficking [314,315,316]. The tight junction proteins are linked to the actin cytoskeleton through scafolding proteins, ZO proteins. ZO-1, ZO-2, and ZO-3 have been identified in ECs [317,318], and their localization and interactions are essential for anchoring the adherens and tight junction protein complexes to the actin cytoskeleton [319,320].

Gap junctions are complexes of intracellular channels that regulate electrical and chemical communication between ECs by allowing direct diffusion of ions and small molecules. The gap junction clusters are formed by hexameric assemblies of tetraspan integral membrane proteins, called connexins (Cx) [321]. The Cx expressions are tissue specific, and vascular ECs have been shown to express Cx37, Cx40, and Cx43 [322,323,324]. Studies have also found that the interruption of gap junctions is involved in BBB dysfunction. Opening of Cx43 hemichannels in ECs were related to hypoxia-induced EC death and vascular leakage [325]. Increased Cx43, gap junction plaque size, and intracellular communication enhanced BBB permeability in mouse CCMs by binding with ZO-1, promoting the dissociation ZO-1 from tight junction complexes [326,327]. In the HHT2 mouse model, reduced Cx40 expression resulted in enlarged arterial vessels, consequently causing blood flow alteration and direct arteriovenous shunts with rarefaction of the capillary network [328].

#### 3.2.4. EC Dysfunctions in bAVMs

Ambiguous vessel identities, a combination of arterial, venous, and capillary features, are shown in bAVMs [43]. Dysregulated arterial (EphB4, ephrin type-B (Ephrin-B) receptor 4) and vein markers (Ephrin-B2) were shown in human and mouse bAVMs [129,138,140,329]. The mechanisms of abnormal EC differentiation were extensively implicated with Notch signaling [129,138,140]. However, a better understanding of how normal EC differentiation and vascular specification are disrupted by specific gene mutations, thereby resulting in bAVM development and a risk for rupture, is required. In addition, there are few studies demonstrating the importance of EC migration in AVMs. For instance, Park et al. showed that defective ALK-1-induced AVMs are associated with impaired flow-induced EC polarization and migration in capillaries and veins, primarily through activation of integrin-VEGFR2 mediated PI3K activation of YAP/TAZ signaling (the full name of TAZ is tafazzin, phospholipid-lysophospholipid transacylase) [246]. *ENG*-deficient ECs showed insensitivity to flow resulting in AVMs, which would be partially due to the activation of PI3K-AKT pathway [249]. In addition, reduced junctional proteins (VE-Cadherin, ZO-1, occludin) were also involved in human bAVMs [38,330]. This evidence suggests that interruption of EC differentiation, polarization and migrations, as well as EC junctions, are essential mechanisms in the vessel remodeling that leads to AVMs. Therefore, further investigation into the mechanisms that drive EC dysfunctions with regard to bAVM pathogenesis should be considered.

### 3.3. Endothelial-to-Mesenchymal Transition (EndMT) and bAVMs

#### 3.3.1. General Definition of EndMT

Endothelial-to-mesenchymal transition (EndMT) is a type of cellular trans-differentiational process in which ECs acquire mesenchymal characteristics, orchestrated through a series of molecular and functional changes. At the molecular level, ECs acquire phenotypic plasticity in EndMT, shedding self-markers, which include VE-cadherin, CD31, Tyrosine kinase with immunoglobulin-like and EGF-like domains-1/2 (Tie-1/2), and von Willebrand factor (vWF), while increasing mesenchymal markers, such as α-smooth muscle actin (α-SMA), CD44, N-Cadherin, vimentin, Snail (known as SNAI1, Snail family transcriptional repressor 1), Slug (also known as SNAI2), TGFβ, and MMP2/9. The ECs undergoing EndMT display spindle-like elongated cell morphology, loss of cell–cell junctions and polarity, and higher motility and invasiveness [331,332,333]. Studies show that EndMT is a dynamic process involving a spectrum of intermediary phases throughout the permanently differentiated mesenchymal cells and intermediate hybrid cells, which express both EC and mesenchymal markers, as well as the ECs that would be reversed from EndMT status [334,335,336]. While EndMT is a critical process in embryonic development, studies demonstrate that EndMT is also significantly associated with certain pathogenetic circumstances, including cancer progression and fibrotic, inflammatory, and vascular disorders [333]. These findings implicate the process of EndMT as a potential therapeutic target in human diseases.

#### 3.3.2. Transcription Modulators of EndMT

EndMT is regulated by numerous transcription factors. Several studies documented the crucial role of the Snail superfamily of zinc finger transcription factors, such as SNAI1, SNAI2, ZEB1, and ZEB2, in EndMT. For example, high expression of endothelial SNAI1 expression was shown to be involved in pathological ocular neovascularization, as well as increased EC morphological changes and motility, which were reversed by the loss of SNAI1. This study also found that the SNAI1 knockdown decreased the expression of numerous genes related to EndMT [337]. Upregulation of Snail was sufficient to induce EndMT, along with inhibition of glycogen synthase kinase 3β (GSK-3β) [338]. Small interfering RNA (siRNA)-suppressed Snail blocked TGF-β-induced EndMT in cultured murine embryonic stem cell-derived ECs [339]. The roles of the other members of the Snail family in EndMT are relatively understudied. However, a reduction in Slug expression was shown to be decrease EndMT in human microvascular ECs [340]. Another study has found that Slug induced VE-cadherin reduction in human dermal microvascular ECs isolated from breast cancer [341]. ZEB1 and ZEB2 induced EndMT, mediating pancreatic cancer or corneal fibrosis [342,343]. Twist-related protein 1 (Twist1) is a member of a large family of helix–loop–helix transcription factors that function as molecular switches to regulate target gene expression [344]. Twist1 overexpression promoted hypoxia-induced EndMT in cultured pulmonary arterial ECs, and mutant *Twist1* failed to induce EndMT [345,346]. EC-specific Twist1-deficient mice confirmed the critical role of Twist1 in EndMT [345]. Twist1 has also been reported to play a role in tissue fibrosis through stimulating EndMT [347,348]. Several other transcription factors, such as sex-determining region Y (SRY)-box 2 (Sox2), the E-26 transformation-specific (ETS) family of transcription factors, and myocardin-related transcription factor (MRTF) have also been identified in relation to EndMT [349,350,351,352]. In addition to transcription factors, extensive evidence shows that micro RNAs (miRNAs) are a critical modulator in EndMT [333]. A study showed that various miRNAs were differetially modulated during TGFβ-induced EndMT [353]. Of these, miR-125b, miR27b, miR-143, miR-21, miR-31, and miR-126a-5p are considered as EndMT inducers [353,354,355,356,357,358,359,360], whereas miR-126, miR-155, miR-200a, miR-18a-5p, and miR-148b seem to have inhibitory roles in EndMT [361,362,363,364,365]. The role of the other non-coding RNAs, such as circular RNAs and long non-coding RNAs, were also implicated in EndMT [359,366,367,368,369]; however, their roles with repect to brain EC function require further investigation.

#### 3.3.3. TGFβ-Induced EndMT

TGFβ signaling is an essential pathway in the EndMT process. Extensive studies have shown that members of TGFβ family of growth factors are the main inducers of EndMT [334,336,338,351,370,371,372,373]. Briefly, an early in vitro study showed that TGFβ1 significantly enhanced α-SMA expression, along with loss of factor VIII antigen (an EC marker) in cultured bovine aortic ECs, which was partially reversible by withdrawal of TGFβ1 [334]. This observation was confirmed by similar studies using bovine aortic and pulmonary arterial ECs treated with TGFβ1 [374,375]. In vivo and in vitro studies revealed that TGFβ2 and TGFβ3 are also potent inducers of EndMT, with TGFβ2 being more prominent and having direct effects on inducing EndMT [376,377]. TGFβ1 and TGFβ3 up-regulated endogenous TGFβ2 expression, and TGFβ2 silencing using siRNA blunted the expression of EndMT markers in TGFβ1- and TGFβ3-treated cells [377]. In addition, in a study on the role of TGFβ in endocardial cell development, *Tgfb1*- or *Tgfb3*-deficient mice did not show any obvious valvular defects, whereas *Tgfb2-*deficient mice displayed multiple defects in atrioventricular cushion formation [378,379]. Furthermore, multiple in vivo studies using mouse or chick models also confirmed that TGFβ2 stimulated EndMT and promoted endocardial cushion and heart development [380]. BMPs have also been found to be involved in EndMT. BMP4 could induce EndMT in human umbilical vein ECs (HUVECs) and cutaneous microvascular ECs (HCMECs), though in an ALK2- and TβRI-dependent manner [381]. Atrioventricular-mayocardium-specific inactivation of BMP2 resulted in endocardial cushion differentiation accompanied by dysregulated EndMT gene expression [382]. Additional studies also showed that deficiency of BMP type I receptors, ALK2 or ALK3, in the endothelium resulted in atrioventricular canal defects via reduced EndMT gene expression [383,384]. With the direct evidence of the role of TGFβ in EndMT, further studies have also revealed the relevance of TGFβ signaling in vascular permeability, cell–cell junctions, and angiogenesis [335,385,386,387].

Canonical pathways of TGFβ-induced EndMT are mediated by Smad-dependent signaling. Binding of TGFβ with its heterodimeric receptor complex (TGF-β receptor types I and II) leads to phosphorylation of Smad2 and Smad3 in the cytoplasm, which form a Smad2/3/4 complex. The Smad2/3/4 complex translocates to the nucleus and binds to TGF-β-responsive genes, regulating EndMT gene expression [333,371,388,389]. Snail and Twist1 regulation were shown to be dependent on this TGFβ-Smad pathway [338,345,390]. Additional evidence has shown that Toll-like receptor (TLR) 5 was additionally involved in the EndMT induced by the TGFβ-Smad-dependent pathway via mediating Snail and Twist expression [391]. In contrast, this TGFβ-Smad pathway can be negatively regulated by inhibitory Smad proteins, Smad6 and Smad7 [392,393]. It is also showed that Sirtuin 1 and 3 and Friend leukemia virus integration 1 (Fli-1) also act as negative regulators of EndMT induced by this canonical TGFβ-Smad pathway [394,395,396]. TGFβ-induced EndMT is also mediated by noncanonical, Smad2/3-independent signaling pathways. For example, activation of the MAPK pathway, including ERK, p38 MAPK, and PI3K and GSK-3 β activation, were required for Snail expression [338]. TGFβ-induced EndMT was also shown to be activated by protein kinase C (PKC)-δ, Abelson murine leukemia viral homolog 1 (c-Abl), or Janus kinase 2 (JAK2) activation, which are distinctive from the Smad pathway [397,398]. These noncanonical pathways may induce the EndMT process in a Smad-independent manner; however, cross-communication between these pathways and Smad signaling, through regulating TGFβ expression, further affect EndMT progress [338,399,400,401].

#### 3.3.4. Other Signaling Pathways Which Induce EndMT

Studies using cultured ECs and animal models found that Notch contributes to EndMT. Noseda et al. confirmed that Jagged1-Notch1/4 interaction down-regulated VE-cadherin and EC markers but enhanced mesenchymal markers and migration in cultured human ECs [402]. Notch1 signaling was shown to induce EndMT in the absence of other EndMT stimuli [403], and Notch-induced EndMT was involved in Slug expression, but not Snail [390,404]. However, studies suggest that Notch synergizes with TGFβ signaling to further increase Snail expression and induces EndMT by interacting with Smad3 [390,405,406]. Interestingly, the TGFβ-Smad2/3-enhanced CXCR7 inhibits Notch activation, subsequently reducing EndMT. This is potentially a feedback mechanism to control the TGFβ-induced EndMT [407]. Using rodent models, studies revealed that Notch-induced EndMT was involved in atrioventricular canal and cardiac cushion development, pulmonary profibrotic myofibroblast development, and pathologic pulmonary fibrosis [403,408,409,410]. However, there is a controversial study which showed that EC-specific inhibition of Notch signaling accelerated EndMT in mice and was accompanied by increased TGFβ1 levels [411].

The WNT proteins are secreted glycoproteins that interact with Frizzled receptors (FZDs), seven-pass transmembrane proteins, on cell surfaces. Through interactions with a variety of co-receptors (e.g., low-density lipoprotein receptor-related protein), WNT/FZD activates diverse intracellular signaling pathways [412,413,414,415,416]. The WNT pathway is mediated by the canonical pathway via the proteins Disheveled (Dsh) and β-catenin, as well as the non-canonical, β-catenin independent, pathways involved in Ca^2+^ signaling [414,416,417]. WNT signaling has been implicated in embryonic development and pathological processes associated with cardiovascular, neurological, inflammatory, and fibrotic diseases [418,419,420,421,422]. Several studies support the role of WNT signaling in EndMT. Using cell lineage tracing, a study showed that canonical WNT/β-catenin signaling induces mesenchymal characteristics in cultured ECs involved in myocardial infarction [418]. Another study demonstrated that inhibition of canonical WNT signaling by blockades of complement receptor antagonists (C3aR/C5aR) prevented expression of EndMT markers and pro-fibrotic proteins in human renal glomerular ECs [423]. An additional recent study also showed that inhibition of WNT signaling via the secreted frizzled-related protein 3 blocks EndMT in the mitral valve after myocardial infarction [424]. The WNT-induced EndMT seems to occur by synergizing with the TGFβ signaling pathway via β-catenin translocation and SMAD-associated transcriptional regulation [333,425,426]. However, there is a controversial study showing that Dickkopf WNT signaling pathway inhibitor 1 (Dkk-1), a WNT inhibitor, enhanced EndMT in aortic ECs [427]. Therefore, the WNT role in EndMT in different cellular and disease contexts needs to be further investigated.

In addition to Notch and WNT signaling, endothelin-1 (ET-1) and caveolin-1 (CAV-1) are also regarded as important EndMT inducers [332,333]. ET-1 is an endogenous vasoconstrictor polypeptide produced by ECs and plays crucial roles in the regulation of multiple vascular functions, as well as the pathogenesis of pulmonary arterial hypertension [428,429,430,431]. Extensive studies have shown that ET-1 can stimulate type I and III collagens and inhibit MMP production, mechanisms associated with fibrotic disease [432,433,434,435,436,437,438,439,440,441]. Another study more directly demonstrated that the EC-derived ET-1 promoted cardiac fibrosis and heart failure through activation of EndMT [442]. While ET-1 alone is potent enough to induce EndMT [443], some evidence suggests that the ET-1-induced EndMT is closely associated with the activation of TGFβ signaling [443,444], which is mediated by ET-1’s autocrine effect on TGFβ1 and TGFβ2 expression [444]. CAV-1 is a member of the caveolin family that is primarily localized to caveolae on the cell membrane [445,446]. The CAV-1 plays a role in EndMT as an inhibitory regulator through internalization, trafficking, and degradation of TGF-β receptors [447,448,449]. One study found that CAV-1-deficiency induced spontaneous EndMT in pulmonary ECs, which was abrogated by the restoration of CAV-1 functional domains [450]. A proteomic analysis study demonstrating that the loss of CAV-1 promotes EndMT in HUVEC cells treated with serum from patients with sepsis also suggests that CAV-1 inhibits the EndMT process [451]. However, the role of ET-1 and CAV-1 in EndMT induction and their interaction with TGF-β signaling should be considered for further investigation.

#### 3.3.5. EndMT as a Pathologic Event in bAVMs

Considering that EndMT would be a critical process in the rise of mesenchymal cells necessary for new vessel formation in developmental and pathologic angiogenesis [331,452,453], recent observations of EndMT-relevant gene and molecular changes suggest that EndMT also plays a key role in the aberrant vessel development and pathology of cerebrovascular malformations. Several studies have reported that increased EndMT markers were observed in cavernous malformations [278,352,454,455,456,457]. Similarly, enhanced EndMT-associated transcription factors (e.g., Snail, Twist, Slug, and Kruppel-like factor 4 (KLF4)) and mesenchymal markers (e.g., αSMA, N-Cadherin, vimentin, actin alpha 2 (ACTA2), and S100 calcium binding protein A4 (S100A4)) were shown in human bAVMs [38,352,458]. Since KRAS mutations were found in bAVMs, some preclinical studies focused on the effect of KRAS in EndMT. Additionally, a series of molecular analyses confirmed EndMT characteristics (i.e., enhanced mesenchymal markers, reduced EC markers, and higher motility) in HUVECs overexpressing KRAS^G12V^ or KRAS^G12D^ [35,279]. Li et al. additionally found several de novo germline mutations in human bAVMs by deep whole-exome sequencing and showed that junction plakoglobin (JUP) and ENG knockdown by siRNAs induced αSMA while decreasing CD31 expression in HUVECs [38]. Therefore, these results suggest that other germline mutations are potentially involved in EndMT and bAVM development, in addition to the well-known role of ENG in HHT-associated bAVMs. EndMT is accompanied by lower expression of junctional proteins, including VE-cadherin, Occludin, and ZO-1, which is associated with bAVM hemorrhagic formation [330].

**Figure 3:**
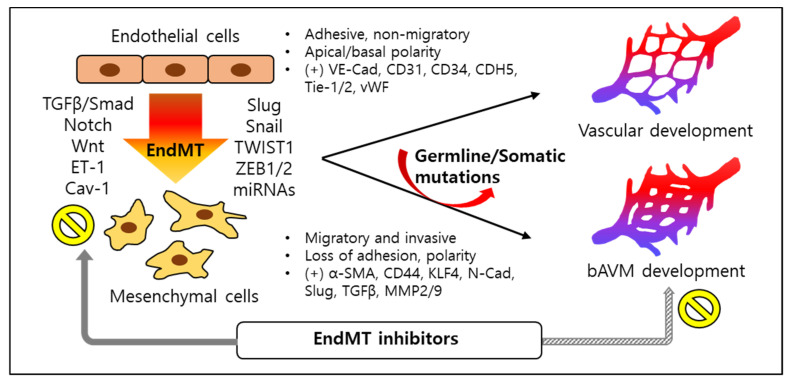
A schematic representation of EndMT involved in bAVMs. The hallmark of EndMT is ECs acquisition of mesenchymal characteristics through diverse molecular signaling, mainly TGFβ signaling. EndMT is a crucial process in vascular development under normal circumstances, by providing ECs as the sources of new vessels and EC mobility; however, EndMT being disrupted by various stimuli (e.g., mutations) contributes to pathologic vessel development, including that of bAVMs. Identification of the exact mechanisms in which bAVM-relevant germline or somatic mutations regulate EndMT and the role of EndMT plays in bAVM pathology will be critical for developing novel therapeutic approaches to treat bAVM patients by targeting of this process.

Because EndMT has recently been explored in bAVMs, the exact molecular mechanisms and functional roles of EndMT in bAVM pathology are largely unknown. So far, few studies have investigated the EndMT signaling mechanisms in the context of bAVMs. For example, a selective MEK1/2 inhibitor, U0126, reversed EndMT in cultured HUVECs, showing the significance of MAPK-ERK signaling in mediating EndMT due to KRAS mutations [38,279]. Furthermore, SMAD4 (a nuclear cofactor of SMAD 2/3) knockdown largely reversed EndMT characteristics in cultured ECs overexpressing KRAS^G12D^ and primary cultured bAVM ECs harboring KRAS mutation. This study demonstrates that activation of TGFβ-BMP-SMAD4 signaling is crucial in the KRAS-mutation-induced EndMT [279]. The inhibitory role of SMAD6 in EndMT and bAVM rupture was also suggested by another study [330]. However, SMAD4 expression and activity were observed to be limited in human brain AVMs with EndMT in the other study [458], suggesting that other signaling pathways are also involved. Accordingly, a study has shown that inhibition of Sox2 signaling stabilized cerebral EC differentiation and lumen formation in matrix Gla protein-null mice, a bAVM model, demonstrating the importance of Sox2 signaling in EndMT and bAVMs [352]. A very recent study reported that exosomal miR-3131 promotes EndMT in KRAS-mutant bAVMs, suggesting miR-3131 as a potential biomarker and therapeutic target in bAVMs with KRAS mutation [459]. Identifying the exact molecular and regulatory mechanisms involved in EndMT and the discovery of specific EndMT inhibitors will be of significant benefit in the development of novel treatments for bAVM patients (Figure 3).

#### 3.3.6. Potential EndMT Inhibitors

Given extensive studies demonstrating the crucial contribution of EndMT to various human pathologies, EndMT is being considered as a novel therapeutic target [460]. Several agents have been identified as potential EndMT inhibitors through the evaluation of various diseases (Table 2). The majority of EndMT inhibitors have been evaluated through their use in cardiac pathology. For example, rapamycin, relaxin, simvastatin, scutellarin, spironolactone, evodiamine, puerarin, and pioglitazone were shown to have efficacy in preventing cardiac fibrosis through preferential targeting of TGFβ signaling over other signaling pathways (e.g., Notch, mTOR, peroxisome proliferator-activated receptor-gamma (PPARγ) [461,462,463,464,465,466,467,468,469]. Others, including losartan and givinostat, were studied for use in myocardial infarctions [470,471,472]. Several other EndMT inhibitors were also evaluated in the context of renal fibrosis and disease, such as sitagliptin, vildagliptin, cinacalcet, and lovastatin [473,474,475,476]. Among them, lovastatin was shown to inhibit KRAS^G12D^-induced EndMT in HUVECs, which is implicated in bAVMs [279]. MEK inhibitor (U0126) was also found to significantly inhibit mutant KRAS-induced EndMT in cultured ECs [38,279], suggesting that inhibition of EndMT via the MEK/ERK blockade could be a mechanism of considerable benefit in bAVM treatment [42,43,151]. Furthermore, there are continuing efforts to discover novel EndMT inhibitors [477]. Therefore, additional studies are required to determine if targeting EndMT using the known or additional novel EndMT inhibitors is a promising approach to treating bAVM patients.

## 4. Conclusions

As we documented in this review, both human and animal studies have identified the causal roles of several germline (e.g., HHT genes) and somatic mutations (e.g., KRAS mutations) in bAVMs. On top of these mutations, studies found additional de novo mutations in some bAVMs, including mutations on *ENG*, *ALK-1*, *SMAD4*, *JUP*, *EXPH5* (encoding exophilin 5), and *EPAS1* (endothelial PAS domain protein 1 encoding hypoxia-inducible factor 2-alpha) genes [19,23,27,28,29,30,31,38], adding greater complexity to the currently understood mechanisms of bAVM pathogenesis. While these de novo mutations, accompanied with the germline mutations, suggest the necessity of bi-allelic loss of the *ENG* or *ALK1* for bAVM genesis [32], the exact roles of the de novo mutations in bAVM pathogenesis are not clear. The number of de novo mutations has been linked to aging [480]. However, de novo mutations found in human bAVMs did not show a significant association with the age of bAVM patients. Most of them were not functional in the bAVM pathology, and only a few of them (e.g., *ENG*, *JUP*, *EXPH5*, *EPAS1*) were shown to have a potential role in bAVM pathophysiology. This study also found that bAVMs with somatic KRAS mutations were prone to rupture, and bAVM patients with certain 16 de novo mutations were found to exhibit epilepsy as the first presenting symptom [38]. These findings suggest that the de novo mutations may be involved in bAVM-associated hemorrhages and complications.

Accumulated evidence clearly demonstrates that ECs play a major role in bAVMs; however, their exact roles and interactions with the other cerebrovascular units are not fully understood. Although this review focused on the fundamental mechanisms of EC dysfunction and EndMT relevant to bAVM pathology, strong consideration should be given towards identifying how mutated ECs interact with and respond to various other influences, including other neurovascular constituents (e.g., mural cells, astrocytes, immune cells) and the extracellular stimuli (e.g., blood flow, inflammatory status, humoral factors, etc.) (Figure 1). While lower mural cell coverages were associated with increased vessel permeability [163,481], several studies have revealed the critical role of signaling pathways such as PDGFB)/PDGFR-β, ANG/TIE2, and EPHRINB2/EPHB4 in regulation of the pericyte and SMC coverage on bAVM vessels, which are well-documented in a review paper by Shabani et al. [63]. Based on the excessive accumulation in human and mouse bAVMs, microglia and macrophages are a key focus in bAVM studies to identify their roles, accompanied signaling mechanisms (e.g., COX2, NFκB, Toll-like receptor (TLR) signaling), and polymorphic analyses on the inflammatory cytokine genes [482]. The roles of other inflammatory cells, such as neutrophils, lymphocytes, and astrocytes, could be linked in bAVM pathophysiology. For example, a higher neutrophil/lymphocyte ratio is correlated with poor outcomes with intracerebral hemorrhages [483]. It is also suggested that neutrophils may play a role in bAVM development through neutrophil extracellular trap (NET) formation and enhancing angiogenesis [484,485,486,487]. Even though they are abundantly present in brain, astrocytes have been given lesser attention in bAVM research; however, the abnormal astrocyte activation seen in human bAVMs suggest their role is linked to bAVM pathology through interaction with the other vascular cells [488]. The abnormally regulated vascular constituents contribute to enhancing circulating inflammatory cytokines/chemokines and reactive oxygen species (ROS) generation, which promotes the systemic influence on vascular permeability in bAVMs through the regulation of junction proteins [489]. In addition, hemodynamic flow is also known to modulate overall EC activity and can influence in the vascular networks via regulation of endothelial gene/protein expression associated with proliferation, migration, inflammatory response, angiogenesis, and vascular integrity [490,491]. As reported, loss of systemic blood flow causes defects in arteriovenous specification in chicks and mice [492,493,494,495]. It has also been reported that hemodynamic stress influences ALK-1 expression and activity, subsequently regulating EC migration towards arteries and vascular diameter regulation through the BMP10-ALK1-SMAD1/5/9 cascade [66,68]. The evidence supports the understanding that a multifactorial set of influences are responsible for bAVM development and progression.

In conclusion, the mechanisms postulated for bAVM development and pathology include complex molecular pathways and cellular functions, as well as extracellular/microenvironment influences. Therefore, comprehensive investigations are needed to identify how these components orchestrate bAVM development and bAVM-associated complications. By further understanding the fundamental functions of each vascular component in physiologic and pathologic circumstances, common mechanisms in vessel malformations of various etiologies can be ascertained, which will enable the development of targeted treatments for bAVM patients.

## Figures and Tables

**Table 1:** Novel agents under recent clinical investigation for AVM-related treatments.

Agent	Target/Mechanism(s)	Stage of Investigation
Rapamycin (*sirolimus*)	mTOR pathway/ Allosteric inhibitor of mTORC1 [188]	- (active) Phase III clinical trial for use in slow-flow AVMs (NCT02638389) [189] - (active) Phase II clinical trial for the treatment of severe/superficial AVMs (NCT02042326) - (completed) Phase II clinical trial showing efficacy treating slow-flow vascular anomalies in children (NCT02509468) [203]
Thalidomide	BBB impairment and angiogenesis/ Immunomodulation, increased mural cell coverage, vessel maturation, and PDGFB expression in ECs [87,204] VEGF antagonism via metabolites [194]	- (completed) Phase II clinical trial showing a reduction in recurrent epistaxis in HHT (NCT01485224) [195] - (completed) Phase II clinical trial investigating effect on GI bleeding in HHT (NCT00389935)
Bevacizumab	VEGF overexpression/ Monoclonal antibody targeting VEGF [205]	- (completed) Phase I clinical trial for treatment of bAVMs. No changes in bAVM volumes reported after 52 weeks. (NCT02314377) [196] - (completed) Phase III clinical trial on treatment of severe bleeding in HHT, showing fewer transfusions were required when compared to placebo (NCT03227263) [206] - (future) Phase II/III clinical trial assessing safety and efficacy in patients with symptomatic bAVMs (NCT06264531)
Pazopanib	Angiogenesis/ Antagonism of VEGF, PDGF, & FGF receptors, as well as c-Kit [200]	- (active) Phase II/III clinical trial investigating effects on epistaxis and anemia in HHT (NCT03850964) - (completed) Phase II clinical trial suggesting efficacy in treating hemangioblastomas in VHL (NCT01436227) [201] - (future) Phase I/II clinical trial assessing efficacy in treating recurrent epistaxis in HHT (NCT03850730)
Trametinib	MAPK/ERK pathway upregulation/ Allosteric inhibitor of MEK 1 & 2 [202]	- (active) Phase II clinical trial evaluating treatment in complicated extracranial AVMs (NCT04258046) - (active) Phase II clinical trial evaluating treatment of AVMs unable to be treated through standard practices [207,208,209] (EudraCT 2019-003573-26) - (active) Phase II pilot study investigating use in surgical unruptured AVMs of the brain and body (NCT06098872)

**Table 2:** Potential EndMT inhibitors.

Indicated Pathway	Agent(s)	Context of Investigation	Clinically Related Outcomes
TGF-β/Smad	Evodiamine [464], Givinostat [470], Losartan [471], MEK inhibitor (U0126) [279], Pioglitazone [463], Prazosin [471], Puerarin [462], Rapamycin [465], Relaxin [466], Scutellarin [468], Simvastatin [478,479], Spironolactone [467]	Cardiac Fibrosis	Reduction in fibroblast activation, type I & III collagen production, pro-fibrotic cytokines production, and increased microvascular density
	Lovastatin [279,473]	KRAS^G12D^-mutated HUVECs [279], Diabetic Nephropathy [473]	Suppression of oxidative stress and glomerular dysfunction
	Liraglutide and Sitagliptin [476]	Renal Fibrosis	Inhibition of glomerular tuft hypertrophy, glomerular mesangial expansion, and microvascular thrombosis
	Vildagliptin and linagliptin [474]	Pulmonary Fibrosis	Inhibited production of pro-fibrotic markers (α-SMA or S100A4) in pulmonary vascular ECs and EndMT cell activity
Undetermined	Cinacalcet [475]	Renal Fibrosis	Prevention of kidney dysfunction, proteinuria, and a reduction in circulating PTH in rodents
	Compound CHIR-99021 [477]	Pulmonary Fibrosis and Chemosensitivity in Irradiated Nonsmall cell lung cancer (NSCLC) cells	Inhibited stress-fiber production and EndMT marker (α-SMA) expression in irradiated cells

## Data Availability

Not applicable.
